# Analysis of the health status and its influencing factors of the low-income populations in Wuxi, China

**DOI:** 10.3389/fpubh.2024.1424448

**Published:** 2024-09-11

**Authors:** Shiming Li, Ying Jiang, Yue Wu, Yingying Ji, Lin Tian, Queping Yang, Haohao Zhu

**Affiliations:** Mental Health Center of Jiangnan University, Wuxi Central Rehabilitation Hospital, Wuxi, Jiangsu, China

**Keywords:** low-income population, physical health condition, influencing factors, Wuxi, China

## Abstract

**Objective:**

To understand the physical health condition and its influencing factors among the low-income population.

**Method:**

Low-income residents who visited or consulted at our Hospital during 2022 were selected for this study. Through telephone or face-to-face interviews, a self-made basic information questionnaire was used for data collection. The physical health level of the low-income population was analyzed, and a logistic regression model was applied to study its influencing factors.

**Results:**

A total of 2,307 people were included in this study, of which 2,069 had various types of diseases, indicating a disease rate of 89.68%. Multivariate logistic regression analysis showed that age ≥ 60 years old (OR = 1.567, 95%CI: 1.122–2.188), poor mental health status (OR = 2.450, 95%CI: 1.203–3.678), smoking (OR = 1.752, 95%CI: 1.269–2.206), pulse pressure difference ≥ 60 (OR = 1.485, 95%CI: 1.164–1.787), and poor hearing (OR = 1.268, 95%CI: 1.026–1.324) were risk factors for disease, whereas being female (OR = 0.729, 95%CI: 0.540–0.984) was a protective factor for physical health.

**Conclusion:**

As a developing country with a large population, we should particularly focus on the physical health issues of the low-income population, take targeted measures for disease situations, and improve the quality of life of the low-income population.

## 1 Introduction

In recent years, with the rapid economic and urbanization development in China, the health issues of the public have gradually received government attention, especially the quality of life and disease conditions of specific groups such as low-income populations. The Chinese government has carried out a series of support and policy support work (1). However, it should also be noted that due to economic reasons, low-income populations often have untreated chronic diseases and are in relatively poor physical health (2). The direct link between low-income and poor health outcomes has been widely documented. Low-income individuals face adverse social factors such as malnutrition, poor living conditions, and discrimination, which contribute to the deterioration of physical and mental health across generations and persistent long-term poverty (3).

This study was conducted in Wuxi City, Jiangsu Province. Wuxi is located between 31°07′ and 32°02′ north latitude and 119°33′ and 120°38′ east longitude, situated in the southeastern part of Jiangsu Province, within the Yangtze River Delta corridor. It borders Suzhou to the east and is 128 km from Shanghai; it faces Huzhou in Zhejiang Province across Taihu Lake to the south. The total area of Wuxi is 4,627.47 sq. km, with the urban area covering 1,643.88 sq. km. In terms of population, by the end of 2023, Wuxi had a registered population of 5.2095 million, with an annual population growth rate of 3.73‰. The urbanization rate of the registered population was 87.56%. The city’s permanent resident population was 7.495 million at the end of the year. Economically, Wuxi’s GDP was 1.55 trillion yuan, with a GDP growth rate of 6%, and a per capita GDP of 206,300 yuan, ranking first among prefecture-level cities nationwide. Although Wuxi’s economic level ranks high nationally and per capita income has increased, it is important to note that the large population and uneven distribution of health and economic resources mean that a portion of the population remains at a low-income level. Therefore, the low-income population should not be overlooked in the pursuit of common prosperity. Currently, China’s low-income groups mainly include manual laborers, lower-tier workers, and other low-income earners. According to data from the second quarter of 2024 regular press conference held by the Ministry of Civil Affairs, the national dynamic monitoring platform for low-income populations covers 80.15 million people, accounting for about 5.7% of the total population. These low-income individuals mainly include nearly 40 million people on subsistence allowances, over 4.6 million people in extreme poverty, and other low-income individuals such as members of families on the edge of subsistence allowances and those being monitored to prevent them from falling back into poverty. To further improve the living conditions of low-income groups, the government and various sectors of society are taking a series of measures. These include strengthening the social security system, raising subsistence allowance standards, and helping low-income groups mitigate disease risks through charitable medical assistance, among other methods.

The health status of China’s low-income groups is influenced by various factors, including working conditions, lifestyle, and dietary habits, which may increase health risks and affect income and medical expenses (4). The health risks faced by low-income groups are becoming an increasing focus of social concern. Moreover, the health status of low-income groups is also affected by other factors. For example, the living conditions of low-income populations in remote areas and the risks they face are complexly intertwined. The vulnerability of their livelihoods and the worsening risk environment can lead to difficulties in living and even poverty. Therefore, conducting surveys on the health status of low-income populations in this region and understanding the related influencing factors to improve their health and living standards remains a top priority for government departments at all levels. Comprehensive measures to improve the health status of this group include strengthening health education and promotion, raising public awareness of health risks, and alleviating their economic pressure and living burdens through policies and social support. This will provide a scientific basis for formulating assistance policies for other gradually developing regions in China.

## 2 Subjects and methods

### 2.1 Subjects

Data were collected from low-income residents who visited or called for consultation at the Wuxi City Central Rehabilitation Hospital. A total of 2,307 cases were collected as research subjects, and the subjects’ basic personal information and disease-related information were collected. Inclusion criteria for research subjects: Age ≥ 18 years old; Able to communicate normally. Researchers with a lot of missing basic information and those who disagreed to participate in the survey were excluded.

### 2.2 Methods and related definitions

Cross-sectional research methods were applied. To ensure the validity and reliability of the self-made questionnaire, it was designed based on the research theme and objectives of this survey. The questionnaire was created to be user-friendly and easy for respondents to complete, thereby improving the completion rate. Additionally, interviews with two senior clinical doctors were conducted, which ultimately led to the finalization of the questionnaire, including gender, age, education level, marital status, smoking, drinking, disease conditions, self-rated mental health status, family care status, height, weight, blood pressure, hearing conditions, etc. Disease condition refers to diseases diagnosed by medical institutions, mainly including history of mental illness, substance dependence, heart attack, hepatitis, cirrhosis, tuberculosis, kidney disease, chronic bronchitis, stroke, hypertension, diabetes, tumor history, coronary heart disease, etc. BMI = weight (kg)/height (m)^2^. Pulse pressure difference is the average value of systolic pressure—diastolic pressure measured three times under resting conditions. Self-rated mental health status refers to whether one feels psychologically healthy. Low-income residents are defined as four types of people: those registered in the civil affairs system as minimum living allowance recipients, extremely poor individuals, families on the brink of minimum living allowance, and families with expenditure difficulties. By testing the reliability and validity of the questionnaire, it was found that the Cronbach’s α coefficient was 0.78 and the average content validity index (S-CVI/Ave) was 0.82, indicating good reliability and validity.

### 2.3 Statistical analysis

A multivariate logistic regression analysis model was constructed using SPSS 20.0 statistical software. Count data were expressed as rates or proportions. Quantitative data that followed a normal distribution were expressed as $\bar{x}\pm s$ . Univariate analysis used the *χ*2 test. Variables with statistically significant differences were included in the logistic regression model. The assignment of independent variables was shown in Table 1. Differences were considered statistically significant at *p* < 0.05.

**Table 1:** Assignment of variables for logistic regression.

Variable	Assignment
Health status	0 = no disease, 1 = with disease
Gender	1 = male, 2 = female
Age	0 = <60, 1 = ≥60
Mental health status	0 = good, 1 = poor
Smoking	0 = no, 1 = yes
Drinking	0 = no, 1 = yes
Pulse pressure difference	0 = <60, 1 = ≥60
Hearing condition	0 = normal, 1 = poor

## 3 Results

### 3.1 Basic situation

A total of 2,307 people were included in the study, 1,568 were males (67.97%) and 739 were females (32.03%). The average age was 47.26 ± 17.64, and the majority had a junior high school education level (52.97%). A total of 2,069 people (89.68%) had illnesses, with the top three diseases being hypertension (50.20%), diabetes (18.25%), and infectious diseases (12.27%).

### 3.2 Univariate analysis of disease status

The disease rate in males was higher than in females, higher in people aged ≥60 years old than in those <60 years old, higher in smokers than in non-smokers, higher in alcohol consumers than in non-alcohol consumers, higher in those with poor mental health than in those with good mental health, higher in those with a pulse pressure difference ≥60 than in those with a pulse pressure difference <60, and higher in those with poor hearing than in those with normal hearing. The differences within each group were statistically significant (*p* < 0.05) (Table 2).

**Table 2:** Univariate analysis of disease status.

Factors	Total number	Number of patients (%)	*χ* ^2^	*p*
Gender			5.638	0.018
Male	1,568	1,423 (90.75%)		
Female	739	646 (87.42%)		
Age			10.231	0.001
<60	1,510	1,332 (88.21%)		
≥60	797	737 (92.47%)		
Education level			2.825	0.243
Elementary and below	623	525 (84.27%)		
Junior high school	1,222	1,119 (91.57%)		
High school and above	456	425 (93.20%)		
Marital status			0.428	0.934
Unmarried	858	746 (86.95%)		
Married	525	461 (87.81%)		
Divorced	893	834 (93.39%)		
Other	31	28 (90.32%)		
Drinking			4.345	0.037
Yes	457	422 (92.34%)		
No	1,813	1,614 (89.02%)		
Smoking			4.839	0.028
Yes	975	890 (91.28%)		
No	1,298	1,148 (88.44%)		
BMI			1.837	0.607
<18.5	75	67 (89.33%)		
18.5–23.9	629	560 (89.03%)		
24–27.9	354	318 (89.83%)		
≥28	140	130 (92.86%)		
Self-assessed mental health			11.444	0.001
Good	1,591	1,404 (88.25%)		
Poor	716	665 (92.88%)		
Family care situation			4.768	0.092
Do not care	105	95 (90.48%)		
Normal	773	709 (91.72%)		
Very concerned	1,241	1,101 (88.72%)		
Pulse pressure			47.423	0.001
<60	2,201	1,965 (89.28%)		
≥60	106	104 (98.11%)		
Hearing status			13.487	0.001
Normal	1,711	1,511 (88.31%)		
Poor	596	558 (93.62%)		

### 3.3 Analysis of influencing factors

The variables that were statistically significant in the univariate analysis were included in the multivariate logistic regression model. The results suggest that the main influencing factors for disease in the low-income population are gender, age, self-rated mental health status, smoking status, pulse pressure difference, and hearing status. Specifically, compared to those under 60 years old, the risk of disease in low-income individual’s ≥60 years old is 1.567 times higher. Those with poor self-rated mental health have a disease risk 2.450 times higher than those with good mental health. Low-income residents who smoke have a disease risk 1.752 times higher than non-smokers. Those with a pulse pressure difference ≥60 have a disease risk 1.485 times higher than those with a pulse pressure difference <60. Low-income individuals with poor hearing have a disease risk 1.268 times higher than those with normal hearing. Being female appears to be a protective factor in the low-income population, with women being less likely to get sick compared to men (Table 3).

**Table 3:** Multivariate logistic regression analysis of influencing factors.

Factors	*β*	Wald *χ^2^*	*p*	OR	95%CI	
					Upper limit	Lower limit
Gender						
Male				1.000		
Female	−0.317	4.267	0.039	0.729	0.540	0.984
Age						
<60				1.000		
≥60	0.449	6.958	0.008	1.567	1.122	2.188
Self-assessed mental health						
Good				1.000		
Poor	0.384	8.381	0.002	2.450	1.203	3.678
Smoking						
No				1.000		
Yes	0.346	4.275	0.037	1.752	1.269	2.206
Drinking						
No				1.000		
Yes	0.187	1.929	0.165	0.829	0.637	1.080
Pulse pressure						
<60				1.000		
≥60	0.555	4.934	0.029	1.485	1.164	1.787
Hearing status						
Normal				1.000		
Poor	0.428	5.675	0.015	1.268	1.026	1.324

## 4 Discussion

Low-income or impoverished populations often experience health damage or loss. The influence of disease impacts a patient’s personal work capacity and can even affect their sources of income (5), intensifying the progression of the disease (6). Through this study, we found that in the Wuxi region, the incidence of various diseases among low-income populations reached 89.68%, a relatively high level and higher than the incidence found in other domestic studies (7, 8). This includes common chronic diseases such as hypertension and diabetes, as well as infectious diseases such as hepatitis B, hepatitis C, and tuberculosis. Therefore, individuals with chronic diseases are more likely to be impoverished due to the disease, creating not only a burden of disease but also significant economic costs (9). Therefore, it is necessary to conduct research investigations based on the health status of the low-income population and its influencing factors, providing further intervention measures.

This study found that women in the low-income population are less likely to suffer from various types of diseases compared to men, possibly because men are more susceptible to disease than women (10, 11). Compared with the low-age group of the low-income population, people aged 60 and older are more likely to develop various chronic or infectious diseases. Research reports that increasing age is a risk factor for chronic diseases (12). As age increases, the body’s immune and physiological recovery abilities gradually decline. Coupled with the impact of economic status, the older adult are more susceptible to various diseases (13). Other research has reported that more older adult people’s households are impoverished (4). We evaluated the mental health status of our study subjects and found that low-income populations who perceive their mental health as poor are more likely to fall ill. This may be because their self-awareness and satisfaction about their mental state are in a poor condition. Poor mental emotions can promote the occurrence and development of diseases, so attention to the mental health status of this population is particularly important (14). Smokers are more likely to develop chronic diseases than non-smokers. Smoking itself produces harmful chemicals like nicotine and tar, which have been proven to be carcinogenic and can also cause chronic lung diseases such as emphysema and bronchitis (15). Research also shows that smoking can exacerbate symptoms of anxiety and depression (16), all of which contribute to the reason why smokers are more prone to chronic diseases. We also found that residents with a pulse pressure difference of ≥60 are more likely to fall ill, it is very likely that an increase in pulse pressure difference will gradually shift from a sub-healthy state to a state of disease, leading to illness (17). We conducted a survey on the hearing status of the low-income population and found that those with poorer hearing are more likely to fall ill. It’s possible that impaired hearing is affected by brain or nerve, mental system abnormalities, which in turn leads to decreased hearing.

Based on this study and Weimer and Vining’s generic policies and policy-related studies (18), combined with the current stage of social development in China, the policy recommendations to improve the health levels of low-income groups mainly include expanding medical insurance coverage, improving the quality of medical services, strengthening health education and disease prevention, and increasing the income of low-income groups. To expand medical insurance coverage, gradually extend the scope of major disease treatment for low-income populations in rural and remote areas, covering major and severe diseases. Further expand the coverage of special treatment programs, incorporating diseases that impose significant local burdens into the local major disease treatment programs, and increase medical assistance efforts for low-income groups (19). This includes improving the medical insurance system, reducing their medical expenses, providing better medical services, and improving the accessibility and affordability of healthcare to benefit more impoverished people. Additionally, encourage social forces to participate and form a diversified health service model. Guide social capital into the medical field to invest in constructing medical institutions or providing medical services. At the same time, leverage the roles of non-governmental organizations and volunteers to provide public health services such as health consultations and check-ups for low-income groups.

Furthermore, strengthen inter-departmental collaboration to form a joint force. Government departments should enhance communication and cooperation to jointly develop and implement health policies for low-income groups. Strengthen cooperation and exchange with international organizations and other countries, learning from their successful experiences and practices to contribute to improving the health levels of low-income groups. Additionally, promote the development of the health industry and encourage the research and production of health products suitable for low-income groups, such as low-cost medicines and medical devices (20). This can not only meet the health needs of low-income groups but also promote innovation and development in the health industry.

In terms of improving medical service quality, all regions should continue to carry out special treatment programs for major diseases among low-income groups, enhance diagnosis and treatment capabilities, and improve medical quality so that most major disease patients can receive better medical services nearby (21, 22). Implement detailed family doctor contracting services for low-income groups, focusing on the standardized management of chronic disease patients such as those with hypertension and diabetes (23). Continuously strengthen health education and disease prevention efforts, advancing the three-year action plan for health promotion in poverty-stricken areas under the guidance of national policies. Conduct health education activities in villages, families, and schools to improve the health levels of residents in impoverished areas (24). Increase support for early diagnosis and treatment projects for major chronic diseases such as cancer in impoverished areas, promoting chronic disease screening, intervention, and early diagnosis and treatment.

To increase the income of low-income groups, increase their wage and business income. Encourage low-income individuals to work in cities or county towns to increase wage income, while developing local specialty industries to provide farmers with more employment opportunities and income sources. Strengthen the construction of the social security system: establishing a sound social security system is fundamental to increasing the income of low-income individuals. The government should increase investment to expand the coverage and level of social security projects such as social assistance, subsistence allowances, medical insurance, and pension insurance. Finally, in terms of education and employment, greater support for low-income groups is also needed. Education is an important means to enhance personal abilities and social status, so investment in the education of children from low-income families should be increased to ensure they receive a good education. Provide vocational training and employment guidance services to help low-income groups improve their employment skills and increase employment opportunities (25), thereby fundamentally improving their economic status and quality of life (26). By seriously implementing these policy recommendations, the government can effectively address the health and economic challenges faced by low-income groups, improve their health levels and quality of life, and promote social equity and harmony.

The advantage of this study is the large sample survey of the health status and influencing factors of low-income groups. However, the study has limitations due to the cross-sectional nature, which restricts causal inference; there are recall and information biases in the survey of selected subjects. Additionally, the lack of relevant data on genetic, psychological, and behavioral factors affects the survey results. Finally, due to the absence of specific income data for low-income groups, it is not possible to conduct a stratified analysis of their economic status, which impacts the stability of the results.

## 5 Conclusion

In summary, in the context of implementing poverty alleviation policies in China, the number of impoverished people is gradually decreasing. However, the health conditions of the impoverished population remain our primary concern. We need to pay attention to the impact of chronic diseases on low-income populations to prevent the reoccurrence of poverty due to illness. Disease health monitoring and economic policy support should be launched for this special population. We should encourage low-income individuals to participate in social labor and provide corresponding policies (27) to help them completely escape poverty. Enhancing the economic income and health knowledge literacy of the impoverished population will ultimately improve their quality of life.

## Data Availability

The original contributions presented in the study are included in the article/supplementary material, further inquiries can be directed to the corresponding authors.
